# A Colonic Lipoma Causing Obstruction: A Case Report and Review of Literature

**DOI:** 10.7759/cureus.50561

**Published:** 2023-12-15

**Authors:** Polyxeni Pichioni, Dimitrios Kokkinovasilis, Stylianos Stylianou, Saant Al Mogrampi

**Affiliations:** 1 Department of Surgery, General Hospital of Imathia, Naousa Health Unit, Naousa, GRC

**Keywords:** colonic pseudo-obstruction, segmental colonic resection, endoscopy, submucosal tumor, colonic lipoma

## Abstract

Colonic lipomas are rare benign lesions of the gastrointestinal tract. They are asymptomatic in the majority of cases. This report aims to present a case involving a 65-year-old female patient reporting rectal bleeding, loss of appetite, and alteration in bowel habits. First, she was submitted to colonoscopy which revealed a sizable ulcerative polypoid lesion in the descending colon, displaying histological characteristics consistent with a hyperplastic polyp and devoid of malignancy indications. An abdominal computed tomography followed which showed diffuse thickening of the colonic wall in the descending colon, resulting in nearly complete luminal obstruction and also a submucosal lipoma measuring 2.6 centimeters at the same location. Laparoscopic intervention ensued, and following a conclusive intraoperative diagnosis of a submucosal lipoma via frozen biopsy, a successful laparoscopic segmental resection of the descending colon with primary anastomosis was executed. Additionally, a comprehensive review of contemporary literature is provided to enhance the understanding of the management approaches applied in analogous cases since established treatment guidelines for colonic lipomas are currently lacking.

## Introduction

Colonic lipomas represent infrequent benign neoplasms of the gastrointestinal tract, with their initial documentation dating back to 1757 by Bauer [1]. Predominantly located in the ascending colon, these lipomas primarily manifest within the submucosal layer, with a mere 10% of cases presenting as multiple lesions [2]. Typically, their occurrence is observed in female individuals during their fifth and sixth decades of life. The majority of colonic lipomas remain asymptomatic, often incidentally discovered through endoscopic procedures, surgical interventions, or computed tomography due to their lack of apparent clinical signs [3]. Lipomas with a diameter greater than 3.5 centimeters are not likely to be asymptomatic. Small asymptomatic lesions only require regular follow-up, whereas larger symptomatic lipomas should be resected [1]. There are a variety of treatment options, including open surgical removal, laparoscopic resection, and endoscopic techniques. Owing to the lack of established guidelines, treatment of colonic lipomas should be individualized and chosen according to local expertise and each patient's medical condition [4]. When there is preoperative exclusion of malignancy and definitive diagnosis of a lipoma, extent of colon resection may be appropriately limited [1]. Without ruling out malignancy, radical surgery with additional lymph node dissection may become necessary.

This case report endeavors to elucidate a unique case involving a lipoma situated in the descending colon, leading to recurrent episodes of bowel obstruction in a female patient. The successful resolution of this case was achieved through laparoscopic segmental colectomy. Additionally, a comprehensive examination of the latest literature is conducted to synthesize the most recent advancements in the management of colonic lipomas.

## Case presentation

A 65-year-old female patient sought outpatient evaluation at the general surgery department of our hospital, reporting alteration in bowel habits, rectal bleeding, and loss of appetite over the past five months. Her medical history included atrial fibrillation and a transient ischemic attack one year prior. She was under medication with acetylsalicylic acid. She had no history of surgeries but had been a heavy smoker for the past three decades. Abdominal examination revealed mild tenderness in the left lower abdominal quadrant, while laboratory test results fell within normal ranges.

**Figure 1:**
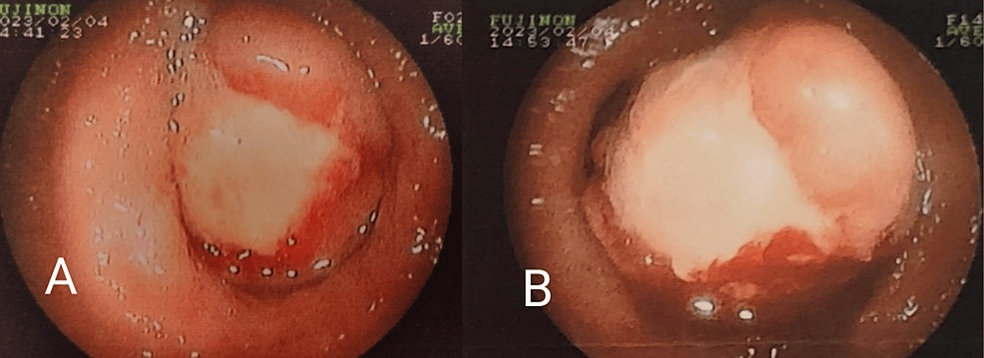
Colonoscopy images. A: A large ulcerative polypoid mass protruding into the colonic lumen at the level of the descending colon. B: Light pressure upon the mass caused its bleeding.

**Figure 2:**
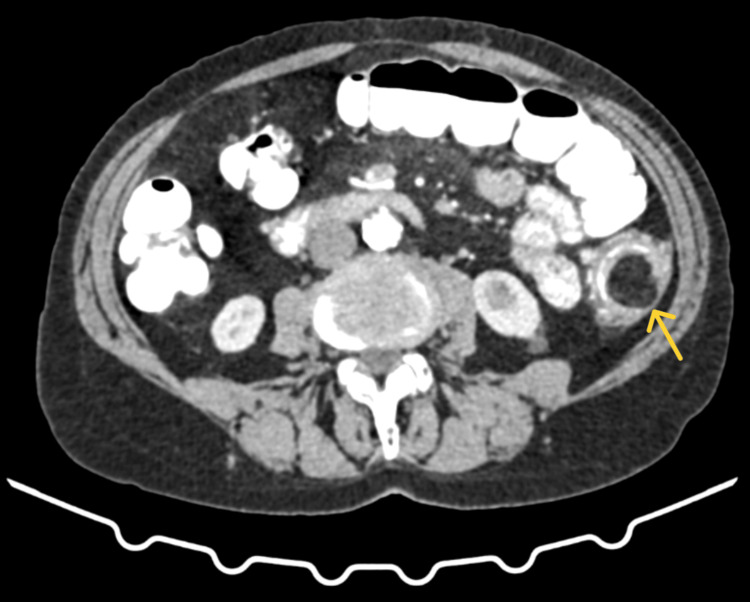
Contrast-enhanced abdominal computed tomography. Axial plane. A submucosal lipoma (yellow arrow) of 2.6 centimeters at the level of the descending colon.

**Figure 3:**
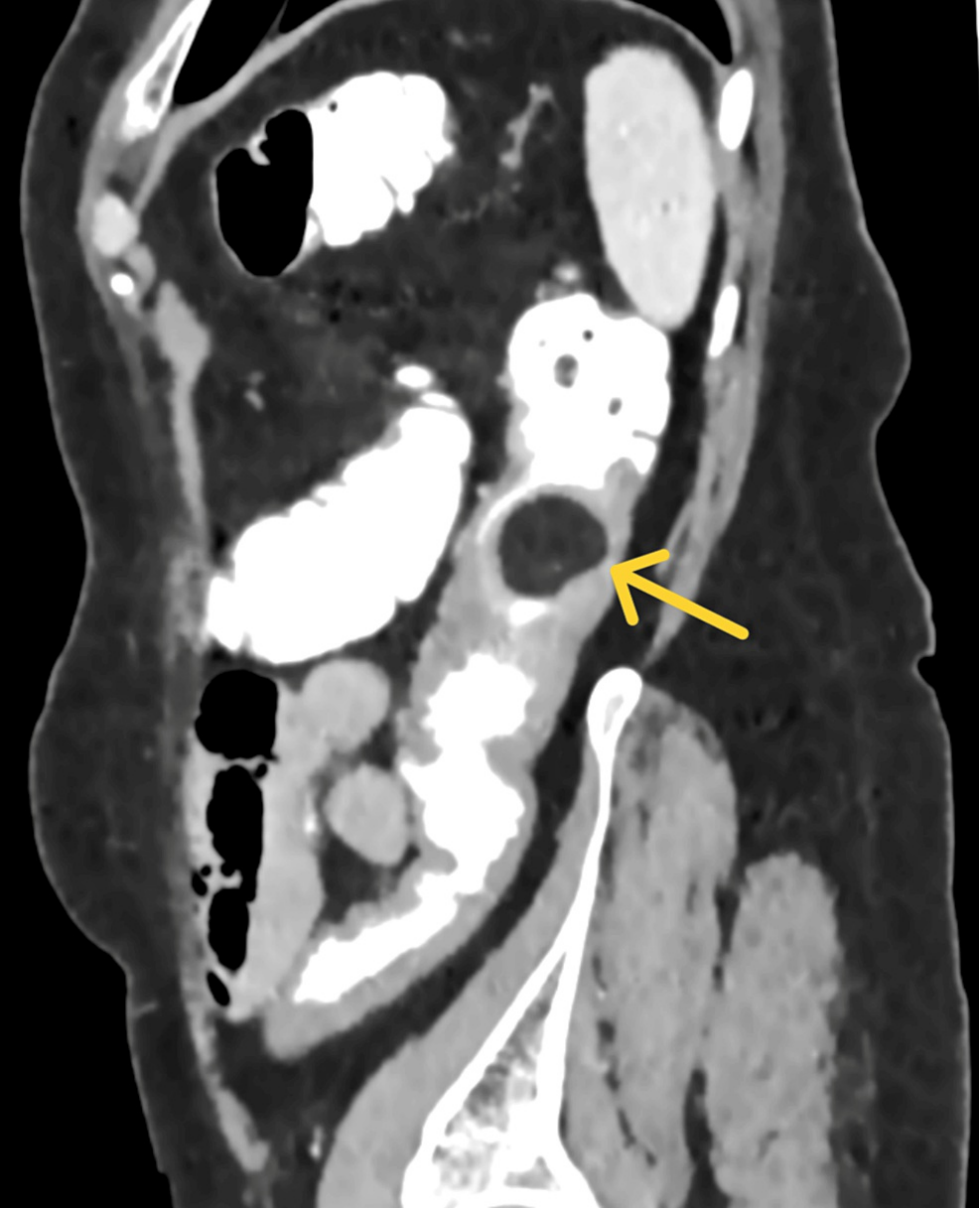
Contrast-enhanced abdominal computed tomography. Sagittal plane. A colonic lipoma (yellow arrow) is causing significant luminal obstruction. Diffuse thickening of the descending colon over a 6-centimeter length.

The patient underwent a colonoscopy a few days before her visit to our department. It revealed a sizable ulcerative polypoid lesion in the descending colon (Figure 1). The lesion was soft and compressible. Biopsy results indicated features consistent with a hyperplastic/inflammatory polyp, with no evidence of malignancy. Subsequent contrast-enhanced abdominal computed tomography disclosed diffuse thickening of the descending colon over a 6-centimeter length, resulting in a significant reduction of the lumen (Figure 2 and Figure 3). Additionally, a tumor of 2.6 centimeters with sharp margin and soft tissue density protruding into the lumen was identified at the same level. Notably, lipomatosis of the ileocecal valve was an unexpected finding of the computed tomography. 

The prevailing consideration for the clinical symptoms of our patient pointed toward a lipoma as the most likely causative factor. A laparoscopic examination was conducted to inspect the affected segment of the descending colon, revealing no free peritoneal fluid or nodules. A colotomy in the colonic wall of the tumor site exposed a yellowish mass, from which a section was extracted for frozen biopsy. The histological features of the lesion in colonoscopy's biopsy were consistent with a hyperplastic polyp. The CT scan findings revealed the presence of a soft tissue density tumor, indicating the presence of a colonic lipoma, so we decided that a specimen from the tumor should be taken intraoperatively for biopsy. The frozen biopsy substantiated the diagnosis of a submucosal lipoma. In a different scenario, we would have chosen a more radical colon resection.

Following the intraoperative exclusion of malignancy, a strategic decision was made to perform a segmental resection of the descending colon. This choice was influenced by the diffuse thickening of the colonic wall at the level of the descending colon and the near-complete obstruction of its lumen. A primary anastomosis of the colon was executed. Subsequent biopsy results affirmed the diagnosis of a submucosal colonic lipoma measuring 3.5 centimeters in diameter, with no indications of malignancy in the surgical specimen. The postoperative course for our patient transpired without complications, leading to her discharge seven days after surgery. A year following the surgical intervention, the patient remains asymptomatic with no recurrence of intestinal symptoms.

## Discussion

The majority of colonic lipomas are typically asymptomatic, and the likelihood of clinical manifestations in colonic lipomas is predominantly associated with the size of the mass rather than its specific location. Lipomas smaller than 2 centimeters are highly improbable to induce symptoms [1]. Colonic lipomas often manifest with mild and atypical symptoms associated with incomplete intestinal obstruction, including nausea, bloating, and intestinal discomfort. Furthermore, instances have been reported where patients sought urgent medical attention, presenting with conspicuous symptoms such as anemia due to gastrointestinal bleeding and even intussusception, a critical surgical emergency [2]. Large colonic lipomas often mimic cancerous lesions due to their infrequency and the complexity of their clinical presentations [3]. Our patient was suffering from rectal bleeding, loss of appetite, and change in bowel movements for five months, so the excision of her colonic lipoma was considered imperative.

Computed tomography scans are considered the best diagnostic tool when it comes to colonic lipomas, while colonoscopies can reveal various signs, such as the cushion sign, that can aid the diagnosis [5]. In our case, although no malignant signs were detected during colonoscopy and the biopsy results indicated a benign lesion, the increase in the thickness of the colon in CT scans prompted apprehension regarding the potential presence of malignancy. However, the submucosal lipoma that was also revealed at the same level was considered the cause of our patient's symptoms, which was confirmed intraoperatively through frozen biopsy. In our review of 18 reported cases of colonic lipomas published in 2023, we summarized patient features and symptoms, lesion characteristics, and the selected treatment plans for each case (Table 1). In contrast to most of these cases, our presented case diverges due to the atypical location of the lipoma at the level of the descending colon, while the majority of the reported lipomas was found in the ascending colon.

**Table 1:** Reported cases of colonic lipomas in 2023 cm: centimeters References: [5-19]

Authors	Age	Sex	Clinical manifestations	Location of lipoma	Size of lipoma	Initial diagnostic method	Selected treatment
Wardeh et al. [5]	48	Male	Abdominal pain intussusception	Transverse colon	Not available	Abdominal ultrasound	Radical colectomy
Niazi et al. [6]	28	Female	Abdominal pain, weight loss, vomiting	Right colon	5.4 cm	Contrast-enhanced abdominal computed tomography	Laparoscopic hemicolectomy
Haider et al. [7]	69	Female	Abdominal pain, constipation	Sigmoid colon	5 cm	Colonoscopy	Loop-and-let-go endoscopic technique
Xie et al. [8]	7	Female	Abdominal pain, intussusception	Transverse colon	5 cm	Abdominal ultrasound	Open surgical removal
Vishnu et al. [9]	53	Male	Abdominal pain, vomiting, intussusception	Ascending colon	5 cm	Contrast-enhanced abdominal computed tomography followed by colonoscopy	Open right hemicolectomy
Vishnu et al. [9]	45	Female	Abdominal pain, vomiting	Ascending colon	8 cm	Contrast-enhanced abdominal computed tomography followed by colonoscopy	Open right hemicolectomy
Bae et al. [10]	78	Male	Asymptomatic	Ascending colon	4.5 cm	Colonoscopy	Endoscopic submucosal dissection
Alwali et al. [11]	70	Male	Acute abdominal pain, sigmoid volvulus	Transverse colon	9 cm	Colonoscopy	Hartman's procedure and endoscopic resection
Akash et al. [12]	40	Male	Intussusception, rectal bleeding	Descending colon	5 cm	Contrast-enhanced abdominal computed tomography followed by colonoscopy	Laparoscopic limited hemicolectomy
Lan et al. [13]	40	Male	Abdominal pain, hematochezia, intussusception	Left colon	6.5 cm	Colonoscopy	Endoscopic ligation
Furuta et al. [14]	65	Female	Asymptomatic	Ascending colon	7 cm	Colonoscopy	Endoscopic submucosal dissection
Nureta et al. [15]	45	Male	Intussusception	Ascending colon	7 cm	Abdominal ultrasound	Open right hemicolectomy
Nureta et al. [15]	60	Male	Intussusception	Ascending colon	4 cm	Abdominal ultrasound	Open right hemicolectomy
Nureta et al. [15]	40	Female	Intussusception	Cecum	6 cm	Contrast-enhanced abdominal computed tomography	Open right hemicolectomy
Phung et al. [16]	63	Male	Abdominal pain, recurrent intussusception	Ascending colon	8 cm	Contrast-enhanced abdominal computed tomography followed by colonoscopy	Endoscopic submucosal dissection
Sui et al. [17]	70	Male	Abdominal pain, intussusception	Ascending colon	5 cm	Contrast-enhanced abdominal computed tomography	Open colectomy
Oura et al. [18]	80	Male	Asymptomatic	Transverse colon	2 cm	Colonoscopy	Endoscopic mucosal resection
Li et al. [19]	49	Female	Abdominal pain, intussusception	Ascending colon	4.5 cm	Contrast-enhanced abdominal computed tomography followed by colonoscopy	Radical right hemicolectomy
Our case	65	Female	Rectal bleeding, intermittent episodes of bowel obstruction	Descending colon	3.5 cm	Colonoscopy followed by contrast-enhanced abdominal computed tomography	Laparoscopic segmental resection

Our case is similar to the case reported by Akash et al. because the authors also performed a laparoscopic limited hemicolectomy with 5-centimeter margin on each side after repeated colonoscopy biopsies that indicated a benign lesion. Additionally, the lesion was also located in the descending colon, as was in our case, and the CT findings disclosed mural thickening suggestive of neoplastic aetiology [12]. In other cases, the authors have decided to conduct radical colectomies because malignancy could not be excluded or the biopsy results showed no specific diagnosis [5,6,9,15,17,19].

Endoscopic methods stand out as the optimal approach for colonic lipoma removal. Various endoscopic techniques, such as snare resection, loop-assisted methods, endoscopic submucosal dissection, and unroofing, have been employed for the removal of large colonic lipomas [4]. However, an analysis of the reviewed cases reveals a prevailing tendency among treating physicians to resort to surgical interventions. Since we did not have the ability to excise the lipoma endoscopically, due to lack of required expertise and equipment, we chose to be as minimally invasive as possible. Thus, we performed a laparoscopic colon resection. Furthermore, we minimized the length of colon resection as much as possible owing to the absence of malignant signs in biopsy results. In only three reported cases, including our case, the lipomas were removed laparoscopically [6,12].

Although the discussion surrounding colonic lipomas and their treatment options is extensive in the literature, clear treatment guidelines remain elusive. Crocetti et al. concluded in their study that laparoscopic surgery represents the gold standard for treating large colonic lipomas or cases where the preoperative exclusion of malignancy is challenging [2]. Conversely, recent studies suggest that unroofing, due to its perceived low-risk nature, should be considered the primary treatment option, particularly for lipomas exceeding 20 millimeters in size or in cases involving patients with concurrent medical conditions [4]. Combining endoscopic treatment methods is also endorsed when initial interventions are insufficient [20]. Despite advancements in endoscopic treatment, colectomies remain the preferred option for addressing these benign lesions in many cases. This inclination prompts contemplation, particularly with regard to cost implications and, more critically, the impact on the morbidity and quality of life of individuals grappling with a condition recognized as benign.

## Conclusions

Colonic lipomas are infrequent and typically asymptomatic, except in instances involving large lesions. Their rarity and diverse clinical presentations often result in misdiagnosis, potentially leading to the unnecessary excision of substantial portions of the colon. Achieving a preoperative diagnosis is challenging, and instances where patients undergo surgery before a definitive diagnosis are not uncommon. Historically, the prevailing perspective suggested that large colonic lipomas necessitated open or laparoscopic surgeries for effective treatment. However, contemporary advances have introduced a range of endoscopic techniques, viable options when the required expertise and equipment are available.
